# Use of an integrated clinical trial database to evaluate the effect of timing of drotrecogin alfa (activated) treatment in severe sepsis

**DOI:** 10.1186/cc4909

**Published:** 2006-05-09

**Authors:** Jean-Louis Vincent, James O'Brien, Arthur Wheeler, Xavier Wittebole, Rekha Garg, Benjamin L Trzaskoma, David P Sundin

**Affiliations:** 1 Department of Intensive Care, Erasme Hospital, Free University of Brussels, Brussels, Belgium; 2 Division of Pulmonary, Critical Care, and Sleep Medicine, The Ohio State University Medical Center, 201 Davis HLRI, 473 West 12th Avenue, Columbus, OH 43210, USA; 3 Department of Medicine, Vanderbilt University, Medical Center North T-1218, Nashville, TN 37232-2650, USA; 4 Department of Intensive Care, St Luc University Hospital, UCL, Avenue Hippocrate 10, 1200 Brussels, Belgium; 5 Lilly Research Laboratories, LCC MC/510/07DC4077 Eli Lilly and Company, Indianapolis, IN 46285, USA

## Abstract

**Introduction:**

Several studies have indicated that early identification and treatment of patients with severe sepsis using standard supportive care improves outcomes. Earlier treatment with drotrecogin alfa (activated) (DrotAA) may also improve outcomes in severe sepsis. Using a recently constructed integrated severe sepsis database, our objectives in this study were to describe the influence of baseline clinical characteristics on timing of DrotAA treatment in patients with severe sepsis, to evaluate the efficacy of DrotAA with respect to timing of administration, and to examine the association between early intervention with DrotAA and patient outcomes, using adjustments for imbalances.

**Methods:**

The database comprises data from 4,459 patients with severe sepsis (DrotAA, *n *= 3,228; placebo, *n *= 1,231) included in five clinical trials conducted in tertiary care institutions in 28 countries. Placebo data came only from randomized trials, whereas data for the DrotAA group came from randomized (PROWESS) and open-label/observational (ENHANCE) trials.

**Results:**

Increased time-to-treatment with DrotAA was significantly associated with more organ dysfunction, greater need of mechanical ventilation, vasopressor use, or recent surgery. Earlier treatment was associated with higher baseline Acute Physiology and Chronic Health Evaluation (APACHE II) scores. Adjusted and unadjusted survival analyses suggested that compared with placebo, DrotAA treatment provided a potential survival benefit, regardless of time to treatment. Survival curves of DrotAA patients treated early compared with those treated late began to separate at 14 days. By 28 days, patients treated earlier had higher survival than those treated later (76.4% versus 73.5%, *p *= 0.03). Sepsis-induced multiorgan dysfunction was the most common cause of death followed by refractory shock and respiratory failure. Modeling of the treatment effect, as a function of time to treatment, suggested increased benefit with earlier treatment.

**Conclusion:**

Using an integrated database of five severe sepsis trials and appropriate statistical adjustments to reduce sources of potential bias, earlier treatment with DrotAA seemed to be associated with a lower risk-adjusted mortality than later treatment. These data suggest that earlier treatment with DrotAA may provide most benefit for appropriate patients.

## Introduction

Severe sepsis is a complex disease associated with high morbidity and mortality. Despite improved understanding of the pathophysiology of severe sepsis and recent advances in supportive care and antimicrobial therapy, severe sepsis remains the leading cause of death in the intensive care unit, and its incidence is increasing [1,2].

Among the many compounds evaluated for the treatment of severe sepsis [3], only drotrecogin alfa (activated) (DrotAA, also known as recombinant human activated protein C) has been shown to reduce mortality in patients with severe sepsis. The pivotal phase 3 placebo-controlled clinical trial PROWESS (Protein C Worldwide Evaluation in Severe Sepsis) demonstrated a 19.4% relative risk reduction in 28-day mortality (6.1% absolute risk reduction) with an increased risk (3.5% versus 2.0%) of serious bleeding events compared with placebo [4]. Subsequently, the global, open-label, single-arm severe sepsis clinical trial ENHANCE (Extended Evaluation of Recombinant Human Activated Protein C) showed similar mortality rates with a somewhat higher rate of serious bleeding events (6.5%) [5]. Recently, an integrated database, the International Integrated Database for the Evaluation of Severe Sepsis and Drotrecogin alfa (activated) Therapy (INDEPTH), of patients receiving either DrotAA or placebo enrolled in five severe sepsis trials with similar entry criteria and conducted by a single sponsor has been constructed and the 'integrated' placebo and DrotAA results have been reported [6]. This large database provides the opportunity for further analyses of primary data from a very large cohort of patients with severe sepsis.

Recent work has shown that the early identification and treatment of patients with severe sepsis using standard supportive care significantly improves outcomes [7]. The ENHANCE trial also suggested greater benefit in patients treated earlier (24 hours or less) than later (more than 24 hours from first documented sepsis-induced organ dysfunction (OD) to treatment) with DrotAA [5]. Using the INDEPTH database, our objectives in the present study were to describe the influence of baseline clinical characteristics on the timing of treatment in patients with severe sepsis, to evaluate the efficacy of DrotAA in patients with severe sepsis with respect to the timing of administration, and to examine the association between early intervention with DrotAA and patient outcomes, using statistical modeling approaches.

## Methods

### The INDEPTH Database

INDEPTH combines primary data of patients with severe sepsis from five Eli Lilly and Company sponsored clinical trials performed between July 1996 and December 2002. All trials were reviewed and approved by the Institutional Review Board at each participating site, and all patients or their designated surrogate signed a written informed consent. A committee of six experts from three countries was organized by the sponsor to review, discuss, and provide recommendations for studies to be included in the integrated database. After three meetings (2003 to early 2004), five trials were identified as appropriate for integration into the database on the committee's recommendations. Ongoing trials were not and could not be considered for inclusion. Trials that ended after the creation of the database were not included for the following reasons: the data became available after the database had been constructed; the patient populations were not similar enough; there was no treatment effect; insufficient data were captured, or a mixture of any or all of the above.

The database incorporates placebo-treated patients from four trials: a phase II DrotAA (Xigris^®^; Eli Lilly and Co., Indianapolis, IN, USA) dosing trial (performed from July 1996 to December 1997 at 40 sites in two countries) [8], the phase III PROWESS trial (performed from July 1998 to June 2000 at 164 sites in 11 countries) [4], and two trials evaluating the efficacy of a secretory phospholipase A_2 _(sPLA_2_) inhibitor [9] (phase II, performed from September 1998 to July 1999 at 72 sites in one country, and phase IIb, performed from October 2001 to October 2002 at 75 sites in five countries). In addition, DrotAA-treated patients from the PROWESS and ENHANCE trials (performed from March 2001 to December 2002 at 400 sites in 25 countries) were incorporated into the database [4,5]. All DrotAA-treated patients in the database received DrotAA at a dose of 24 μg kg^-1 ^h^-1 ^for 96 hours. Only the PROWESS trial contributed patients who received DrotAA and patients who received placebo (all sites and countries contributed patients to both placebo and treatment groups). The remaining trials either were not placebo-controlled (the ENHANCE trial; all sites and countries contributed patients to the treatment group), or had a treatment therapy other than DrotAA at 24 μg kg^-1 ^h^-1 ^(namely the sPLA_2 _trials; only placebo patients were used, and all sites and countries contributed patients to the placebo group), or had DrotAA administered at a variety of doses (DrotAA phase II dosing trial; only placebo patients were used, and all sites and countries contributed patients to the placebo group). All patients in the five trials received supportive care at the discretion of the investigator.

Inclusion criteria were very similar across trials and, in brief, consisted of the following: infection, presence of at least three criteria of the systemic inflammatory response syndrome, and at least one OD (in the phase IIb sPLA_2 _trial, this was at least two ODs). Exclusion criteria in the phase II DrotAA dosing, PROWESS, and ENHANCE trials included patients at high risk of bleeding or likely to die from non-sepsis-related causes within 28 days. Only patients at high risk of death from non-sepsis-related causes within 28 days were excluded from the sPLA_2 __2 _trial, patients had 36 hours or less to meet inclusion criteria, then 6 hours or less to begin study drug infusion (patients had to have at least one OD within 24-hour period before inclusion); for the Phase IIb/sPLA_2 _trial, patients had 48 hours or less to meet inclusion criteria and begin study drug infusion (patients had to have at least three systemic inflammatory response syndrome criteria within 48 hours of study drug infusion and the presence of at least two ODs within 24 hours from the onset of the first OD).

### Statistical analyses

Time to treatment was defined as the interval between the first documented sepsis-induced OD and the administration of DrotAA or placebo. Logistic regression and Cox regression analyses were used to estimate odds and hazard ratios of 28-day mortality associated with DrotAA versus placebo treatment at increasing durations of time to treatment (continuous data). Logistic regression analyses provided point estimates related to a landmark endpoint at 28 days, whereas Cox regression provided hazard ratios describing the entire survival experience over the 28-day follow-up period. These analyses were adjusted for age and Acute Physiology and Chronic Health Evaluation (APACHE) II score, as well as a propensity score (used in observational studies to account for differences that occur between treatment groups in non-randomized comparisons) to adjust for the non-randomized nature of the data. The values of major interest in these models correspond to the interaction between treatment and time to treatment. In addition to the above propensity score for predicting treatment group, we also built a separate propensity for the timing of treatment that was not included in our final models because it did not significantly add to the value of the models. Random effects for protocol (trial) were also considered but did not result in statistically nonzero variances and were removed before the final fitting of our models. Random-effects models were constructed with Proc NLMIXED in SAS version 8.2 (SAS, Cary, NC, USA).

In addition, for hypothesis-generating purposes, 24 hours was an empirically defined duration for the time-to-treatment analyses for purposes of simple tabular presentation. Patients were split into two groups: those starting infusion of DrotAA or placebo within 24 hours of first documented OD (time to treatment 0 to 24 hours) and those starting infusion of DrotAA or placebo after 24 hours of first documented OD (time to treatment 24 hours or more). These patients were combined across trials for final analyses. For model building, time to treatment was used as a continuous variable, to retain as much information as possible about timing in producing model estimates.

The univariate influence of baseline characteristics on the time to treatment was estimated with separate linear regressions with each baseline variable (independent variable) and time to treat as a continuous variable (dependent variable). Joint modeling on time to treatment was also performed with stepwise selection, with alpha values of 0.05 for entry and 0.10 for retention. Although of necessity it was assumed that all important baseline determinants of outcome were measured, it is understood that this is unlikely, if not impossible.

## Results

There were a total of 4,459 patients in the INDEPTH database, for 4,456 of whom 28-day mortality data were available. The difference resulted from three patients who were discharged from the hospital and subsequently lost to follow-up. Of the patients with mortality data, 3,225 were patients receiving DrotAA and 1,231 were patients receiving placebo.

Baseline characteristics of patients from the INDEPTH database are presented in Table 1 and overall were relatively well balanced. DrotAA patients had more ODs and need for vasopressors but they also had somewhat lower APACHE II scores. As shown in Table 2, baseline differences between therapy groups were observed for ethnicity and APACHE II score for both the treated early (0 to 24 hours) and late (more than 24 hours) groups. It is likely that much of the difference in APACHE II score was influenced by the ENHANCE trial, which had uncharacteristically low APACHE II scores compared with other measures of disease severity, and a longer enrollment period [5]. Differences in number of ODs and vasopressors were also observed for the more than 24 hours group. Although other statistically significant differences were observed, they may have little clinical significance.

The influence of baseline characteristics on the timing of treatment was explored by using univariate analyses. As demonstrated in Table 3, for patients with more sepsis-induced ODs, patients on mechanical ventilation or vasopressors, or patients with a recent surgery, time to treatment was significantly increased. For example, patients who were receiving mechanical ventilation were 'treated' 5.4 hours later than patients who were not receiving mechanical ventilation, after their first documented sepsis-induced OD. There was a positive and direct correlation between time to treatment and these baseline characteristics. As the number of ODs increased, or patients were receiving mechanical ventilation or vasopressors, or had recent surgery (probably a result of protocol instruction to commence or resume DrotAA infusion only 12 hours or more after surgery), the time to treatment increased. In contrast, patients with higher APACHE II scores (defined by quartiles) were treated significantly earlier after their first documented sepsis-induced OD. For APACHE II scores there was an inverse relationship between time to treatment and this baseline characteristic. As APACHE II scores increased, the time to treatment decreased. Neither age nor sex had a significant effect on time to treatment, although there may have been a trend toward males being treated later. These potential predictors of time to treatment were also fitted jointly in a multivariable model and produced similar *p *values and estimates to those in univariate analyses. The exception was baseline vasopressor use, which became insignificant after the inclusion of baseline ventilator use (data not shown).

**Figure 1:**
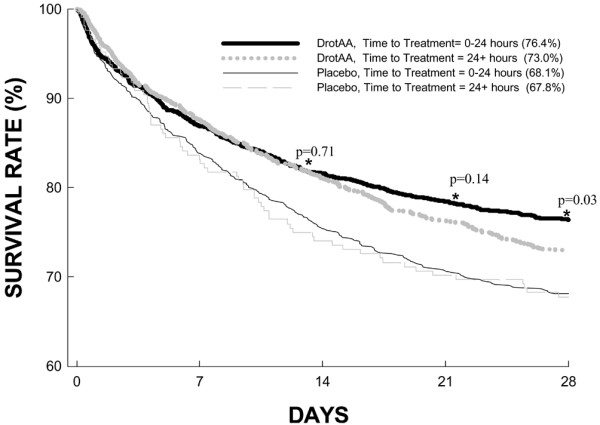
INDEPTH survival curves for placebo-receiving and DrotAA-treated patients by time to treatment. The percentage 28-day survivals are shown parenthetically in the key. Kaplan-Meier survival curves are displayed for therapy groups (namely DrotAA and placebo), as well as for time-to-treatment groups (namely 0 to 24 hours and more than 24 hours). Both DrotAA time-to-treatment curves were significantly different from the placebo time-to-treatment curves. At 14 days, the DrotAA earlier treatment curve (0 to 24 hours) started to diverge from the later treatment curve (more than 24 hours). The difference between the DrotAA earlier and later treatment curves was significant at 28 days (*p *= 0.03). DrotAA, drotrecogin alfa (activated).

Kaplan-Meier 28-day survival curves for patients receiving placebo and DrotAA by time to treatment are presented in Figure 1. Unadjusted and adjusted analyses suggested that there was a potential survival benefit associated with DrotAA treatment, compared with placebo, regardless of time to treatment (only unadjusted analysis is shown). Although the two DrotAA curves were not significantly different during the first two weeks, they began to diverge at 14 days. The difference between the treated early (0 to 24 hours) and the treated late (more than 24 hours) curves became significant by 28 days. Patients treated earlier with DrotAA (0 to 24 hours) had a significantly higher 28-day survival (76.4%) than patients treated later with DrotAA (more than 24 hours; 73.5%) at day 28. No significant timing-related differences were observed in the placebo survival curves (0 to 24 hours, 68.1%; more than 24 hours, 67.8%).

Potential differences in the types of death that patients experienced, based on whether they were treated early (0 to 24 hours) or late (more than 24 hours), were explored and are displayed in Table 4. Because in most cases the number of events was small, we do not provide statistical values and report the data from an observational perspective. The types of death were categorized by treatment group (DrotAA versus placebo), early (days 1 to 14) and late (days 15 to 28) deaths, and by time to treatment (0 to 24 hours, and more than 24 hours). Deaths from sepsis-induced multiorgan dysfunction (MOD) were most common, whether they occurred early or late (days 1 to 14 and days 15 to 28). Deaths from refractory shock and respiratory failure comprised most of the rest of the deaths. Regardless of treatment group or time to treatment, 'late' refractory shock deaths were approximately half that of 'early' refractory shock deaths. In contrast, deaths from respiratory failure increased with time (days 1 to 14 versus days 15 to 28) regardless of treatment group or time to treatment.

There was little, if any, difference in sepsis-induced MOD deaths between DrotAA patients treated earlier (0 to 24 hours, 44.4%) and later (more than 24 hours, 41.9%) during the period 1 to 14 days (see Table 4). However, there seemed to be a considerable difference between those patients treated earlier (34.0%) than later (50.5%) during the period 15 to 28 days. In contrast, placebo patients 'treated earlier' had fewer sepsis-induced MOD deaths than those 'treated later' during the period 1 to 14 days (43.6% versus 56.6%). Although there were too few events during the period 15 to 28 days to produce a reasonable estimate, there were numerically fewer deaths in placebo patients 'treated' later.

**Figure 2:**
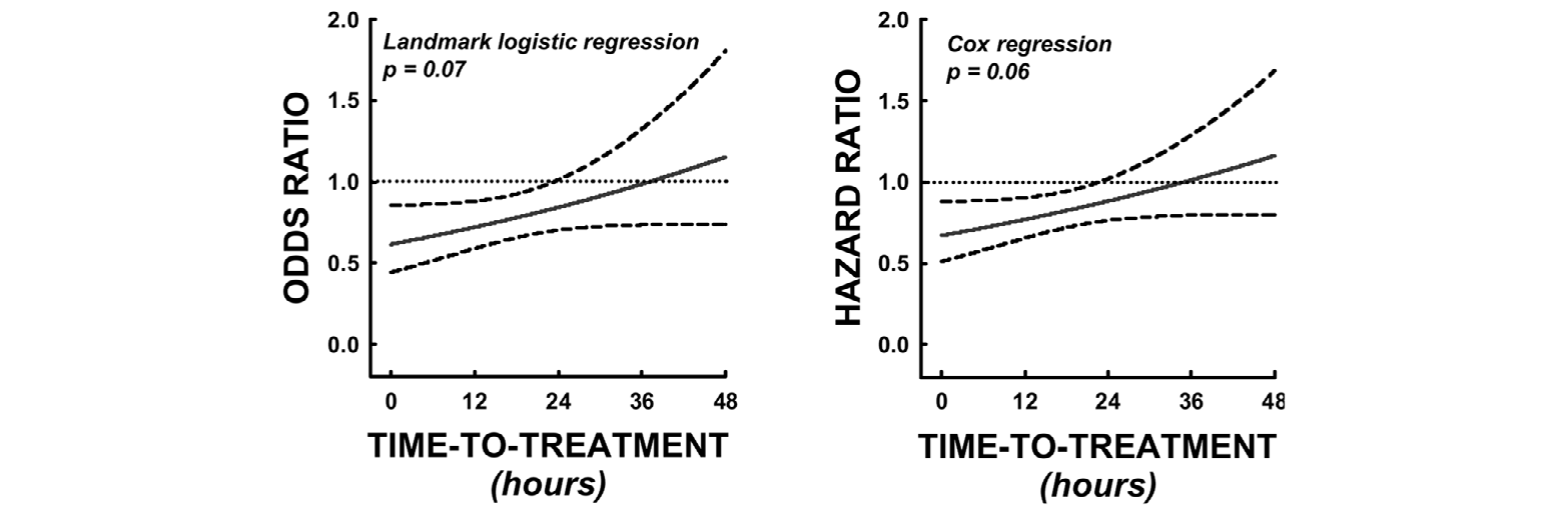
Landmark logistic and Cox regression analysis of DrotAA treatment effect by time to treatment. With the use of logistic (odds ratios for DrotAA versus placebo) and Cox (hazard ratios for DrotAA versus placebo) regression analyses, modeling of the treatment effect as a function of time to treatment was performed. In both analyses there was a trend (logistic regression, *p *= 0.06; Cox regression, *p *= 0.07) toward a more beneficial effect with earlier administration of DrotAA (solid line, ratios less than 1 until 36 hours). The most precise estimates of the model were between 12 and 24 hours, as indicated by the narrowest 95% confidence intervals (dashed lines). Outside this range, estimates of benefit were much less precise (that is, wider 95% confidence intervals furthest from the solid line). DrotAA, drotrecogin alfa (activated).

Lastly, modeling of the treatment effect was performed as a function of time to treatment. Results from the 28-day landmark logistic regression (mortality odds ratios for DrotAA versus placebo at day 28) and Cox regression (mortality hazard ratios for DrotAA versus placebo for the whole 28-day period) analyses are presented in Figure 2. As indicated by the solid line, in both analyses there was a trend toward a more beneficial effect with earlier administration of DrotAA (odds and hazard ratios less than 1 until about 36 hours). The most precise estimates of the model were between 12 and 24 hours (narrowest 95% confidence intervals shown as dashed lines). On either side of this period, estimates were less precise because smaller numbers of patients were treated beyond 24 hours, indicated by the wider 95% confidence-interval lines. In the adjusted model, logistic (28-day landmark) regression analysis suggested that treatment with DrotAA within 24 hours of OD was associated with lower odds of death (23%), compared with treatment after 24 hours. Furthermore, adjusted Cox regression analysis (whole 28-day period) suggested that earlier treatment was also associated with a lower hazard of death (19%) for the 28 days of follow-up.

## Discussion

In this analysis of an integrated database of five clinical studies in severe sepsis, the use of DrotAA was associated with reduced mortality, regardless of the timing of treatment. This suggests that therapy with DrotAA in patients with severe sepsis is beneficial, even after OD has been present for more than 24 hours. Data were sparse for treatment times of more than 36 hours after sepsis-induced OD, and therefore caution must be used in making conclusions about the benefit of DrotAA at later times. However, there seemed to be a trend toward improved outcomes among patients treated earlier with DrotAA. Such a trend was not observed among the comparative placebo patients. These data suggest that the association between the timing of treatment was due to earlier treatment with DrotAA rather than to earlier identification of severe sepsis.

Unlike most meta-analyses of clinical trials, the INDEPTH database allows the review of patient-level data. This permits greater risk adjustment to account for the non-randomized nature of the study. In assessing an effect of the time of treatment with DrotAA, a data set such as this is essential. For example, in the PROWESS study [4], the average time from initial OD to infusion of study drug was 17.5 hours. Only 11% of PROWESS patients began DrotAA infusion more than 24 hours after OD, which limits the power of an analysis with only the subjects from this trial. Therefore, pooling this trial with other studies with similar inclusion and exclusion criteria permits an examination that is not otherwise feasible.

To account for the differences between these studies, a variety of statistical techniques were employed. A previously published propensity score was used to adjust for the non-randomized nature of the use of DrotAA [6]. In addition, a second propensity score was used to adjust for covariates associated with the time to treatment. Ultimately, there was a persistent independent association between earlier treatment with DrotAA and outcome. With the adjusted model (adjusted for covariates and propensity scores), logistic regression analysis suggested that treatment within 24 hours of OD with DrotAA was associated with 23% lower odds of death at 28 days, compared with treatment more than 24 hours after sepsis-induced OD. Also in the adjusted model, Cox regression survival analysis suggested that earlier treatment was associated with a 19% lower adjusted hazard of death for the first 28 days of follow-up. Given the data, these trends suggest an association between earlier treatment with DrotAA and improved outcomes.

Although this cohort provided considerable opportunities for novel analyses, there were limitations to the study. Despite the use of several statistical approaches to adjust for differences between subjects in the various studies, this was not a single randomized trial. Any adjustment was therefore limited to measured covariates. In addition, the results might have been skewed by the number of patients from individual studies [5] and the relatively small number of placebo patients in comparison with DrotAA-treated patients. The lack of placebo patients might be of particular importance at later times, where even less information was available. However, we were encouraged by the similarities in baseline measures among the subjects in the various studies. In addition, the mortality rate among placebo patients was very similar across the studies. These similarities were reassuring and suggest that the study cohort was relatively homogeneous. Although nonlinear models were fitted to the data, they did not provide additional insight or value and were therefore not included.

A limitation that could not be addressed by statistical methods alone was a potential bias from differences in the natural progression of severe sepsis. Most subjects treated with DrotAA more than 24 hours after OD came from a single study [5]. Because this study was not placebo controlled, it is likely that those receiving therapy later were enrolled in the study because of their failure to improve without DrotAA. Supporting such a possibility, in the ENHANCE study, patients treated later were more likely to require vasopressor agents (76% versus 71%) and mechanical ventilation (88% versus 75%) than those treated within 24 hours of OD [5]. This could bias the results toward a benefit for earlier treatment. Additionally, those patients treated within the first 24 hours might have had care providers more attentive to the signs of sepsis and potentially more attentive care overall. However, extensive risk-adjustment was used, including the use of propensity scores for the probability of early treatment and treatment with DrotAA. These statistical adjustments should reduce the potential for bias in the results.

Through this analysis we were able to identify patient factors associated with the time to treatment (see Table 3). The need for mechanical ventilation or vasopressors, additional baseline ODs, and recent surgery were independently associated with a longer time to treatment with DrotAA. This might reflect the fact that initial efforts to stabilize a patient (such as appropriate antibiotic therapy or fluid resuscitation) were performed before the use of DrotAA was initiated. It might also reflect the use of APACHE II scores to direct therapy (see below). Although not confirmed by prospective studies, the potential importance of the timing of drug administration observed here suggests that efforts to incorporate the use of DrotAA into early treatment protocols for severe sepsis might serve to hasten treatment and improve outcome. Several efforts, such as the recently published guidelines for the treatment of severe sepsis [10], promote the idea of early identification and treatment of patients with severe sepsis in accordance with evidence-based guidelines.

An additional factor that might have delayed treatment among more severely ill patients is that these studies required consent for subject inclusion. Sicker patients are less likely to have the capacity to provide informed consent. The need to locate surrogates, inform them of the seriousness of the illness, and obtain proxy consent may delay study entry and drug administration.

Interestingly, whereas other measures of baseline illness severity seemed to increase the time to treatment (namely ventilator use or vasopressor use), APACHE II points shortened the interval between OD and drug administration. APACHE II is a more global assessment of risk, including age, chronic health status, and acute physiology score. Of these parameters, the acute physiology score may decrease with increasing time, as a result of supportive care (namely resuscitation), which can normalize several abnormalities (such as hypotension, metabolic acidosis, and sodium abnormalities). This highlights the potential weakness in using APACHE II to assess disease severity in the context of drug administration in the intensive care unit. The delay in treatment among patients with recent surgical procedures probably reflects the evaluation of the bleeding risk in the early postoperative period, with a protocol requirement that patients be more than 12 hours after surgery for inclusion.

Using a time-to-event or survival analysis, we found that the benefit of earlier treatment with DrotAA did not become apparent until after 14 days and was not statistically significant until 28 days after study entry. The separation in survival curves between those treated with DrotAA and those receiving placebo occurred much earlier. This suggests that the benefit of DrotAA over placebo is apparent early. However, earlier versus later DrotAA therapy has an additional effect that was not observed until 14 days after DrotAA treatment was started. An explanation for this observation may reside in differences in the causes of death in severe sepsis. Sepsis-induced MOD was the most common cause of death throughout the 28 days of follow-up (see Table 4). However, earlier treatment (within 24 hours of OD) with DrotAA seemed to attenuate the number of deaths due to sepsis-induced MOD, during days 15 to 28. This difference accounted for a majority of the difference in overall mortality seen between the group receiving earlier DrotAA treatment and the group receiving this therapy later. The comparative placebo group did not have a similar association. It is possible that early treatment with DrotAA (within 24 hours of OD) does not change the course of septic shock or respiratory failure in isolation but has a more pronounced effect on the resolution of ODs in the form of MOD.

This study suggests that treatment with DrotAA within 24 hours may carry a larger survival advantage for patients with severe sepsis, compared with those treated more than 24 hours after OD. However, later treatment with DrotAA was also associated with lower mortality when compared with patients receiving placebo. Because of the burden of disease and the expected increase in the number of cases of severe sepsis, there is an emphasis on improving the early identification of severe sepsis. Recent studies showing promise in affecting the outcome of patients with severe sepsis involve early intervention. In a study of early goal-directed therapy of patients with sepsis, the study protocol was begun an average of 1.4 hours after arrival in the emergency department [7]. The largest study of corticosteroid supplementation in severe sepsis required drug administration within 8 hours of hypotension [11]. Other evidence supports the use of medical emergency and shock teams to rapidly identify and treat patients with sepsis [12]. The finding in the present study that earlier administration of DrotAA to appropriate patients may have greater benefit than later therapy fits into this paradigm. As sepsis progresses, the pathophysiologic profile may change and be less amenable to intervention. The initial pro-inflammatory state is replaced by a condition of relative immunosuppression [13]. Better defining the phase of illness that a septic patient occupies might improve our ability to tailor care for each patient. As it becomes possible to better characterize the stage of sepsis for an individual patient, it is still likely that early intervention will be an effective strategy. By preventing the progression to later stages of sepsis and shortening the duration of OD, patients will be at lower risk for iatrogenic complications and secondary nosocomial infections.

## Conclusion

By combining records from several clinical studies of severe sepsis conducted by a common sponsor, the INDEPTH database permits an analysis of severe sepsis therapy with the use of patient-level data. In this data set, earlier treatment with DrotAA, within 24 hours of OD, was associated with lower risk-adjusted mortality than later treatment (more than 24 hours after OD). A similar time-to-treatment effect was not observed in patients receiving placebo. Although not confirmed prospectively, these data suggest that earlier treatment with DrotAA may provide the most benefit for appropriate patients.

## Key messages

• 	Analysis of data from 4,459 patients with severe sepsis from five clinical trials in an integrated database showed that increased time to treatment with drotrecogin alfa (activated) was associated with more organ dysfunction, greater need of mechanical ventilation, and greater use of vasopressors.

• 	Moreover, early treatment (within 24 hours of appearance of first organ dysfunction) with drotrecogin alfa (activated) was associated with a lower risk-adjusted mortality than later treatment.

• 	These data suggest that early treatment with drotrecogin alfa (activated) in appropriate patients may carry the greatest benefit.

## Abbreviations

APACHE = Acute Physiology and Chronic Health Evaluation; DrotAA = drotrecogin alfa (activated); ENHANCE = Extended Evaluation of Recombinant Human Activated Protein C; INDEPTH = International Integrated Database for the Evaluation of Severe Sepsis and Drotrecogin alfa (activated) Therapy; MOD = multiorgan dysfunction; OD = organ dysfunction; PROWESS = Protein C Worldwide Evaluation in Severe Sepsis; sPLA_2 _= secretory phospholipase A_2_.

## Competing interests

Eli Lilly and Co. provided funding for this study. J-LV, JO'B, XW, and AW have all participated in clinical trials sponsored by Eli Lilly and Co. J-LV and AW have served as paid consultants for Eli Lilly and Co. RG, BLT, and DPS are employees and stockholders of Eli Lilly and Co.

## Authors' contributions

J-LV and DPS conceived and designed the study. J-LV, JO, AW, and XW participated in the individual studies included in the integrated database and contributed to data collection. J-LV, JO, BLT, RG, and DPS conducted the principal analysis and drafted the manuscript. All authors contributed to revision of the manuscript. All authors read and approved the final manuscript.

## Figures and Tables

**Table 1:** Overall INDEPTH baseline patient characteristics

Parameter	Placebo (*n *= 1,231)	DrotAA (*n *= 3,228)	*p*
Age, years (mean ± SD)	60.3 ± 16.5	59.5 ± 17.0	0.15^a^
<65 (%)	55.2	54.5	0.84^b^
65 – 74 (%)	24.2	25.1	
≥75(%)	20.6	20.5	
Male sex (%)	57.6	57.6	0.99^b^
Caucasian ethnicity (%)	79.4	88.3	<0.0001^b^
APACHE II score (mean ± SD)	24.6 ± 7.8	22.7 ± 7.5	0.0001^a^
<25 (%)	53.2	60.4	<0.0001^b^
≥25(%)	46.8	39.6	
Number of ODs (mean ± SD)	2.4 ± 1.1	2.6 ± 1.1	<0.0001^a^
1 (%)	20.6	18.1	0.07^b^
≥2(%)	79.4	81.9	
Time from first OD to start of infusion (mean ± SD)	23.9 ± 111.4	23.8 ± 13.6	0.97^a^
Mechanical ventilation (%)	78.3	79.7	0.31^b^
Vasopressors (%)	64.1	70.2	0.0002^b^
Recent surgery (%)	33.1	35.1	0.24^b^

APACHE, Acute Physiology and Chronic Health Evaluation; DrotAA, drotrecogin alfa (activated); INDEPTH, International Integrated Database for the Evaluation of Severe Sepsis and Drotrecogin alfa (activated) Therapy; OD, organ dysfunction. ^a^Student's *t *test; ^b^χ^2 ^test.

**Table 2:** INDEPTH baseline patient characteristics based on time to treatment

Parameter	Time to treatment (h)				Between-treatment *p*	
	Placebo (*n *= 1,175)		DrotAA (*n *= 3,216)			
	0 – 24 (*n *= 967)	>24 (*n *= 208)	0 – 24 (*n *= 1,882)	>24 (*n *= 1,334)	0 – 24 h	>24 h
Age, years (mean ± SD)	60.2 ± 16.5	60.5 ± 16.0	58.9 ± 17.8	60.2 ± 15.7	0.05^a^	0.82^a^
<65 (%)	54.8	57.2	53.8	55.4		
65 – 75 (%)	24.7	21.6	25.2	25.0		
>75 (%)	20.5	21.2	21.0	16.6		
Male sex (%)	56.3	62.0	55.8	60.1	0.83^b^	0.60^b^
Caucasian ethnicity (%)	80.4	76.4	87.1	90.0	<0.0001^b^	<0.0001^b^
APACHE II score (mean ± SD)	24.9 ± 7.8	24.1 ± 7.9	23.4 ± 7.6	21.8 ± 7.3	<0.0001^a^	<0.0001^a^
<25 (%)	52.2	53.8	57.2	64.9		
≥25(%)	47.8	46.2	42.8	35.1		
Number of ODs (mean ± SD)	2.4 ± 1.1	2.6 ± 1.1	2.5 ± 1.3	2.8 ± 1.1	0.26^a^	0.01^a^
1 (%)	21.2	17.8	23.0	11.3		
≥2(%)	78.8	82.1	77.0	88.7		
Time from first OD to start of infusion (mean ± SD)	15.7 ± 6.1	35.8 ± 12.4	15.1 ± 6.2	35.5 ± 7.9	0.01^a^	0.75^a^
Mechanical ventilation (%)	76.6	85.6	73.9	87.6	0.11^b^	0.41^b^
Vasopressors (%)	63.8	67.0	67.2	74.6	0.08^b^	0.02^b^
Recent surgery (%)	32.1	36.5	31.1	40.8	0.41^b^	0.50^b^

Total numbers of patients do not add up to those in Table 1 because of missing time-to-treatment data (44 had missing data, 23 had time-to-treatment values of more than 72 hours, and 1 had a time-to-treatment value of less than 0). APACHE, Acute Physiology and Chronic Health Evaluation; DrotAA, drotrecogin alfa (activated); INDEPTH, International Integrated Database for the Evaluation of Severe Sepsis and Drotrecogin alfa (activated) Therapy; OD, organ dysfunction. Statistical comparisons are between DrotAA and placebo patients treated in 0 to 24 and more than 24 hours: ^a^Student's *t *test; ^b^χ^2 ^test.

**Table 3:** Influence of baseline characteristics on duration of time to treatment based on univariate analyses

Patient variable	Time to treatment (h)	*p *(univariate)
Age (years)		0.15
<65	22.6 ± 12.3	
65 – 74	22.3 ± 11.9	
≥75	22.0 ± 11.4	
Sex		0.08
Male	22.7 ± 12.1	
Female	22.0 ± 11.9	
APACHE II score		< 0.0001
First quartile (3 – 17)	23.9 ± 13.4	
Second quartile (18 – 22)	22.9 ± 12.5	
Third quartile (23 – 28)	21.9 ± 11.2	
Fourth quartile (29 – 53)	20.9 ± 10.7	
Number of organ dysfunctions		< 0.0001
1	19.0 ± 12.2	
2	21.7 ± 11.8	
3	23.6 ± 11.7	
4	23.9 ± 11.5	
5	27.0 ± 12.7	
On a ventilator		< 0.0001
Yes	23.5 ± 12.0	
No	18.1 ± 10.9	
On vasopressors		< 0.0001
Yes	23.3 ± 11.7	
No	20.9 ± 12.8	
Recent surgery		< 0.0001
Yes	24.4 ± 11.8	
No	21.3 ± 12.0	
Unknown	24.4 ± 13.2	

Results are means ± SD. APACHE, Acute Physiology and Chronic Health Evaluation score. Statistics are based on one-way analysis of variance.

**Table 4:** Types of death by treatment, time period, and time to treatment

Type of death	DrotAA				Placebo			
	Days 1 – 14 (*n *= 600)		Days 15 – 28 (*n *= 204)		Days 1 – 14 (*n *= 287)		Days 15 – 28 (*n *= 84)	
	0 – 24 h (*n *= 347)	>24 h (*n *= 253)	0 – 24 h (*n *= 97)	>24 h (*n *= 107)	0 – 24 h (*n *= 234)	>24 h (*n *= 53)	0 – 24 h (*n *= 71)	>24 h (*n *= 13)
Sepsis-induced multi-organ dysfunction, % (*n*)	44.4 (154)	41.9 (106)	34.0 (33)	50.5 (54)	43.6 (102)	56.6 (30)	32.4 (23)	23.1 (3)
Refractory septic shock, % (*n*)	27.1 (94)	25.3 (64)	12.4 (12)	13.1 (14)	26.1 (61)	20.8 (11)	12.7 (9)	15.4 (2)
Respiratory failure, % (*n*)	8.7 (30)	10.7 (27)	22.7 (22)	16.8 (18)	13.7 (32)	13.2 (7)	18.3 (13)	15.4 (2)
MI or primary cardiac arrhythmia, % (*n*)	5.8 (20)	6.7 (17)	10.3 (10)	5.6 (6)	3.4 (8)	1.9 (1)	2.8 (2)	15.4 (2)
Hemorrhage, % (*n*)	4.0 (14)	2.4 (6)	2.1 (2)	0.9 (1)	0.4 (1)	None	None	15.4 (2)
Other, % (*n*)	10.1 (35)	13.0 (33)	18.6 (18)	13.1 (14)	6.8 (16)	3.8 (2)	29.6 (21)	15.4 (2)

Other types of death include stroke and unknown causes. DrotAA, drotrecogin alfa (activated); MI, myocardial infarction.
